# Regeneration mechanisms in Syllidae (Annelida)

**DOI:** 10.1002/reg2.98

**Published:** 2018-04-14

**Authors:** Rannyele P. Ribeiro, Christoph Bleidorn, M. Teresa Aguado

**Affiliations:** ^1^ Departamento de Biología Facultad de Ciencias Universidad Autónoma de Madrid Cantoblanco 28049 Madrid Spain; ^2^ Animal Evolution and Biodiversity Georg‐August‐Universität Göttingen 37073 Göttingen Germany; ^3^ German Centre for Integrative Biodiversity Research (iDiv) Halle‐Jena‐Leipzig 04103 Leipzig Germany

**Keywords:** annelid, epimorphosis, fission, morphallaxis, schizogamy

## Abstract

Syllidae is one of the most species‐rich groups within Annelida, with a wide variety of reproductive modes and different regenerative processes. Syllids have striking ability to regenerate their body anteriorly and posteriorly, which in many species is redeployed during sexual (schizogamy) and asexual (fission) reproduction. This review summarizes the available data on regeneration in syllids, covering descriptions of regenerative mechanisms in different species as well as regeneration in relation to reproductive modes. Our survey shows that posterior regeneration is widely distributed in syllids, whereas anterior regeneration is limited in most of the species, excepting those reproducing by fission. The latter reproductive mode is well known for a few species belonging to Autolytinae, Eusyllinae, and Syllinae. Patterns of fission areas have been studied in these animals. Deviations of the regular regeneration pattern or aberrant forms such as bifurcated animals or individuals with multiple heads have been reported for several species. Some of these aberrations show a deviation of the bilateral symmetry and antero‐posterior axis, which, interestingly, can also be observed in the regular branching body pattern of some species of syllids.

## 1 INTRODUCTION: OVERVIEW OF ANNELID REGENERATION

Regeneration is a postembryonic morphogenesis characterized by the ability of an organism to restore lost parts of the body (Bely & Nyberg, 2010; Brockes & Kumar, 2008). Regeneration is widely distributed among animals and occurs at the level of cells, tissues, internal organs, structures, and even the whole body (Bely & Nyberg, 2010; Pfeifer, Dorresteijn, & Fröbius, 2012). Annelids, commonly referred to as “segmented worms,” have remarkable abilities of whole‐body regeneration, even from few segments (Bely, 2006, 2014; Seaver, 2003). Hence some species have been considered as excellent models to investigate regeneration, such as *Alitta virens* (Bakalenko, Novikova, Nesterenko, & Kulakova, 2013; Kozin, Filippova, & Kostyuchenko, 2017)*, Capitella teleta* (Hill, Ferkowicz, & Grassle, 1988; Jong & Seaver, 2017), *Cirratulus cirratus* (Weidhase, Bleidorn, & Helm, 2014), *Enchytraeus japonensis* (Myohara, Yoshida‐Nora, Kobari, & Tochinai, 1999; Ogawa et al., 2017), *Eurythoe complanata* (Müller, Berenzen, & Westheide, 2003; Weidhase, Bleidorn, Beckers, & Helm, 2016), *Lamellibrachia satsuma* (Miyamoto, Shinozaki, & Fujiwara, 2014), *Platynereis dumerilii* (Prud'homme et al., 2003; Starunov, Voronezhskaya, & Nezlin, 2017), *Pristina leidyi* (Bely & Wray, 2001; Nyberg, Conte, Kostyun, Forde, & Bely, 2012; Özpolat, Sloane, Zattara, & Bely, 2016)*, Timarete punctata* (Weidhase, Helm, & Bleidorn, 2015), and *Typosyllis antoni* (Aguado, Helm, Weidhase, & Bleidorn, 2015; Weidhase, Beckers, Bleidorn, & Aguado, 2016; Weidhase, Beckers, Bleidorn, & Aguado, 2017).

For annelids, two different processes leading to regeneration are distinguished: epimorphosis and morphallaxis (Kozin et al., 2017; Özpolat et al., 2016). Epimorphosis is characterized by the activity of somatic stem cells, dedifferentiation, and re‐differentiation processes, resulting in the appropriate re‐establishment of tissue polarity, structure, and form of the organism (Agata, Saito, & Nakajima, 2007; Alvarado & Tsonis, 2006; Bely & Nyberg, 2010; Özpolat & Bely, 2016). The dedifferentiation stage is represented by the formation of a blastema, a tissue that contains undifferentiated cells and acts as a growth zone (Agata et al., 2007; Boilly, Faulkner, Jobling, & Hondermarck, 2017). In contrast, blastema formation is absent in morphallaxis, and the remaining part of the body remodels drastically acquiring morphologies consistent with new positional identities or maintaining normal proportions of the body (Agata et al., 2007; Özpolat & Bely, 2016).

Several studies in annelids have compared juvenile development with regeneration, since both processes (may) involve the establishment of a proliferation zone, also known as a segmental addition zone (SAZ) (Balavoine, 2014; Zattara & Bely, 2016). Additionally, developmental pathways of key transcriptional factors can be triggered by regenerative processes (Bakalenko et al., 2013; Novikova, Bakalenko, Nesterenko, & Kulakova, 2013). However, during early development the body axes are established de novo, while during regeneration body axes are defined by the remaining old tissue (Boilly, Boilly‐Marer, & Bely, 2017). Thus, a different mechanism might be used for establishment of body axes in regenerating individuals (Boilly, Boilly‐Marer et al., 2017).

**Figure 1:**
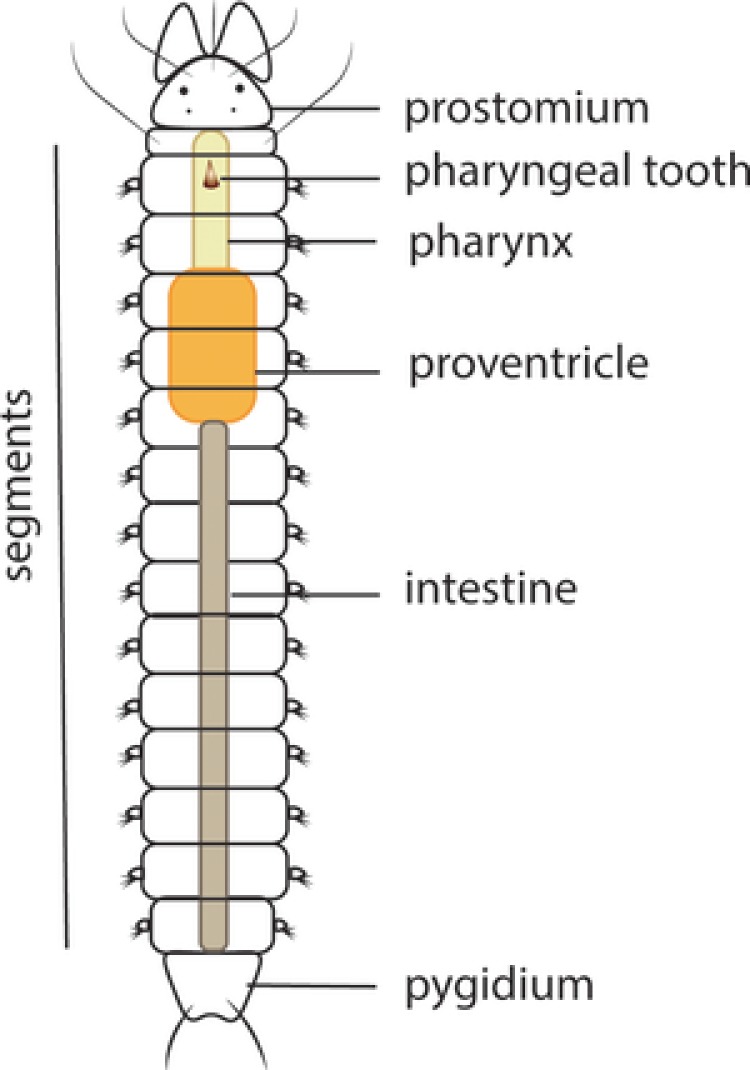
Schematic drawing of the syllid body plan. Illustration of the main features in the morphology of a syllid: prostomium, segments, pygidium and digestive tube components, pharynx, pharyngeal tooth, proventricle, and intestine

The annelid body consists of a prostomium (the anterior cap or head), a set of segments which are often equipped with chaetae‐bearing parapodia (body wall projections used for locomotion), and a pygidium (the posterior cap or tail) (Figure 1). In annelids, posterior regeneration is found widely distributed across taxa, whereas anterior regeneration is restricted to fewer groups (Hyman, 1940). It has been supposed that anterior regeneration is ancestral for annelids, and subsequently this ability has been lost independently across the annelid tree (Bely, Zattara, & Sikes, 2014; Zattara & Bely, 2016). The addition of segments by the action of a SAZ is clearly detectable during posterior regeneration (Balavoine, 2014). Observations of anterior regeneration show an antero‐posterior developmental gradient of newly forming segments, in which the anterior cap (prostomium) forms first, and the SAZ is not clearly detectable or transient.

**Figure 2:**
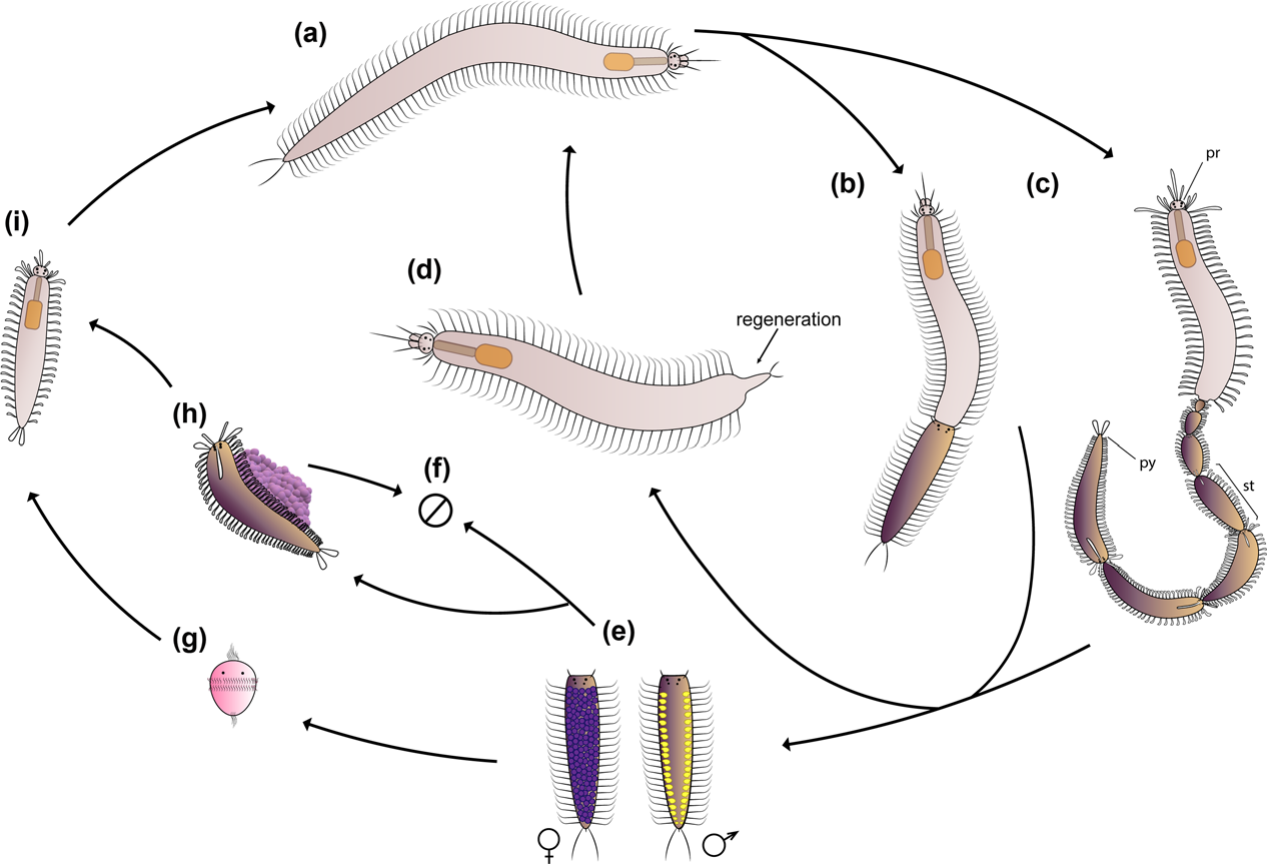
Schematic representation of schizogamy in syllids. (A) Adult individual of a schizogamous species. Schizogamy can occur by scissiparity or gemmiparity. (B) Scissiparous individual during stolonization, based on *Syllis* spp. (Franke, 1999). (C) Gemmiparous individual during sequential gemmiparity based on *Myrianida pachycera* (Okada, 1933b). When the stolons are completely developed, they are detached for spawning. The stock animal regenerates the posterior body after the detachment of stolons. (D) Individual during posterior regeneration. (E) Male and female stolons perform spawning for exchange of gametes; scheme based on stolons of *Syllis* spp. (F) After spawning, stolons die. (G) Larval development in non‐brooding species; larvae disperse in ocean. (H) Alternatively, autolytine female stolons live during external brooding and development of the embryos; mature female carries an egg sack until the release of juveniles; ventral brooding; based on *Myrianida pachycera* (Okada, 1933b). (I) Growth of juveniles until mature stage to start a new stolonization process. pr, prostomium; py, pygidium; st, stolon

Additionally, the regenerative processes in invertebrates are closely related to agametic reproduction (Brockes & Kumar, 2008; Zattara & Bely, 2016). Several species, such as *Pristina leidyi* (Bely & Wray, 2001; Özpolat & Bely, 2015; Özpolat et al., 2016; Zattara & Bely, 2011) and *E. japonensis* (Myohara, Niva, & Lee, 2006; Myohara et al., 1999; Yoshida‐Noro, Myohara, Kobari, & Tochinai, 2000), reproduce asexually in a process that involves regeneration. Furthermore, groups such as Eunicida and Syllidae have reproductive modes (schizogamy or stolonization) that include stages of segment regeneration in the posterior body (Aguado, Glasby, Schroeder, Weigert, & Bleidorn, 2015; Aguado, Helm et al., 2015; Franke, 1999; Rouse & Pleijel, 2006) (Figure 2, Table 1).

**Table 1:** Features of regeneration in Syllidae: sexual reproduction, resegmentation and literature review

		Anterior resegmentation			Posterior resegmentation			
Species	Reproduction	Prt	Time	N seg	Pyg	Time	N seg	References
*Amblyosyllis formosa*	Epigamy	NA	NA	NA	4	−	7	Abeloos, 1952
*Epigamia alexandri*	Epigamy	−	−	−	−	−	−	Malaquin, 1893; Saint‐Joseph, 1886
*Epigamia macrophtalma*	Epigamy	NA	NA	NA	−	42	10	Allen, 1927a
*Myrianida edwardsi*	Schizogamy	−	−	2	5	−	13	Okada, 1929; Saint‐Joseph, 1886
*Myrianida pinnigera*	Schizogamy	−	−	−	−	−	>20	Dehorne, 1917; Okada, 1935
*Proceraea picta*	Schizogamy	−	−	8	−	−	11	Allen, 1927a, 1927b; Okada, 1929, 1934
*Proceraea scapularis*	Schizogamy	−	−	−	−	−	−	Okada, 1933a
*Procerastea halleziana*	Schizogamy	4	39	29	3	39	35	Allen, 1923, 1927a; Caullery, 1925
*Eusyllis assimilis*	Epigamy	−		9	−	−	−	Malaquin, 1893
*Eusyllis blomstrandi*	Epigamy	−	53	4	−	−	−	Boilly, 1961
*Odontosyllis fulgurans*	Epigamy	−	−	6	−	−	−	Okada, 1929; Saint‐Joseph, 1886
*Odontosyllis ctenostoma*	Epigamy	−	−	−	−	−	−	Michel, 1909b; Schroeder & Hermans, 1975
*Synmerosyllis lamelligera*	Epigamy	−	−	−	−	−	−	Allen, 1927b; Okada, 1929
*Exogone naidina*	Epigamy	−	−	−	−	−	−	Pagenstecher, 1862; Viguier, 1884
*Alcyonosyllis aidae*	Schizogamy	−	−	−	−	−	−	Álvarez‐Campos et al., 2013
*Branchiosyllis cirropunctata*	Schizogamy	−	−	−	−	−	−	Michel, 1909b, 1909c
*Haplosyllis spongicola*	Schizogamy	−	−	2	−	−	18	Albert, 1887; Okada, 1929; Saint‐Joseph, 1886; Wissocq, 1966
*Opisthosyllis brunnea*	Schizogamy	−	−	2	−	−	3	Okada, 1929
*Parahaplosyllis brevicirra*	Schizogamy	−	−	−	−	−	−	San Martín, Hutchings, & Aguado, 2010
*Parahaplosyllis kumpol*	Schizogamy	−	−	−	−	−	−	Álvarez‐Campos et al., 2013
*Ramisyllis multicaudata*	Schizogamy	−	−	−	−	−	−	Aguado, Glasby et al., 2015; Glasby et al., 2012; Schroeder et al., 2017
*Syllis amica*	Schizogamy	5	70	4	9	70	20	Boilly, 1962a, 1962b, 1965a, 1965b, 1967a, 1967b, 1967c, 1967d; Durchon, 1951; Verguer‐Bocquet, 1979
*Syllis armillaris*	Schizogamy	−	−	4	−	−	−	Malaquin, 1893; Saint‐Joseph, 1886
*Syllis gracilis*	Schizogamy	8	−	18	−	−	8	Boilly & Thibaut, 1974; Mesnil, 1901; Mesnil & Caullery, 1919
*Syllis hyalina*	Schizogamy	−	−	2	−	−	−	Malaquin, 1893; Durchon, 1959
*Syllis prolifera*	Schizogamy	35	40	0	7?	−	18	Franke, 1980, 1983, 1986; Franke & Pfannenstiel, 1984
*Syllis rosea*	Schizogamy	−	−	2	−	−	−	Langerhans, 1879
*Syllis variegata*	Schizogamy	−	−	2	−	−	−	Andrews, 1892; Langerhans, 1879; Malaquin, 1893; Saint‐Joseph, 1886
*Syllis vittata*	Schizogamy	−	−	−	−	−	−	Durchon, 1959; Michel, 1909b
*Trypanosyllis krohnii*	Schizogamy	−	−	−	−	−	−	Marion & Bobretzky, 1875; Michel, 1909a
*Trypanosyllis zebra*	Schizogamy	−	4	5	−	−	24	Delye, 1962; Junqua, 1957; Michel, 1909b
*Typosyllis antoni*	Schizogamy	4	14	6	4	18	16	Aguado, Helm et al., 2015; Weidhase, Beckers et al., 2016; Weidhase et al. 2017
*Typosyllis pigmentata*	Schizogamy	*−*	60	3	−	−	10	Heacox & Schroeder, 1982

Prt,: time for prostomium appearing, in days; Time, maximum time of resegmentation, in days; N seg, maximum number of regenerated segments; Pyg, time for pygidium appearing, in days. NA, not applicable. Dashes represent lack of data.

Syllidae is an interesting group for studying regeneration. It is one of the largest groups within Annelida in terms of biodiversity, with a wide variety of reproductive modes and different regenerative processes (Aguado, Nygren, & Siddall, 2007; Franke, 1999; Okada, 1929). Syllids occur in all marine benthic habitats and about 700 species are currently known, classified in five families, Anoplosyllinae, Autolytinae, Eusyllinae, Exogoninae, and Syllinae, and some independent groups, such as *Amblyosyllis* and *Perkinsyllis* (Aguado & San Martín, 2009; Aguado, San Martín, & Siddall, 2012; Aguado et al., 2007). They are easily identified to the family level by the presence of the proventricle, a differentiated element of the digestive tube, which can be considered as a synapomorphy of the group (Figure 1) (Aguado et al., 2007, 2012; Fauchald & Rouse, 1997; Franke, 1999; Glasby, 1993; Okada, 1929).

Studies about syllid regeneration are limited and inhomogeneous in terms of investigated species, processes, and applied methods. Nevertheless, the available information represents a treasure trove for future investigations. The purpose of this study is to summarize available data on regeneration in this group of animals. We focus on the regenerative abilities and reproductive modes of syllids, differences between anterior and posterior regeneration, cells and tissues involved, and aberrant forms as a result of anomalous regenerative processes.

## 2 SYLLID REGENERATION

Most studies dealing with regeneration in syllids were published at the end of the 19th century (Langerhans, 1879; Malaquin, 1893; Marion & Bobretzky, 1875) and during the 20th century (Allen, 1923; Boilly & Thibaut, 1974; Delye, 1962; Durchon, 1959; Franke, 1980; Heacox & Schroeder, 1982; Junqua, 1957; Michel, 1909b; Okada, 1929; Wissocq, 1966). The first experiment under laboratory conditions was conducted with the species *Syllis gracilis* by Mesnil (1901). Following studies were focused mainly on the relationship between regeneration and sexual reproduction based on experiments of removal of the pharynx and/or proventricle, which might play a role during stolonization (Durchon, 1957, 1959; Junqua, 1957; Okada, 1938; Wissocq, 1966, 1970a). Some of the most recent studies were those published by Franke (1980, 1983), Heacox and Schroeder (1982), and Franke and Pfannenstiel (1984). After three decades, this topic re‐emerged with a study about the role of the proventricle during the stolonization of *T. antoni* (Weidhase, Bleidorn et al., 2016). This species was also used for investigating muscular and nervous system regeneration (Weidhase, Beckers et al., 2016; Weidhase et al., 2017).

**Figure 3:**
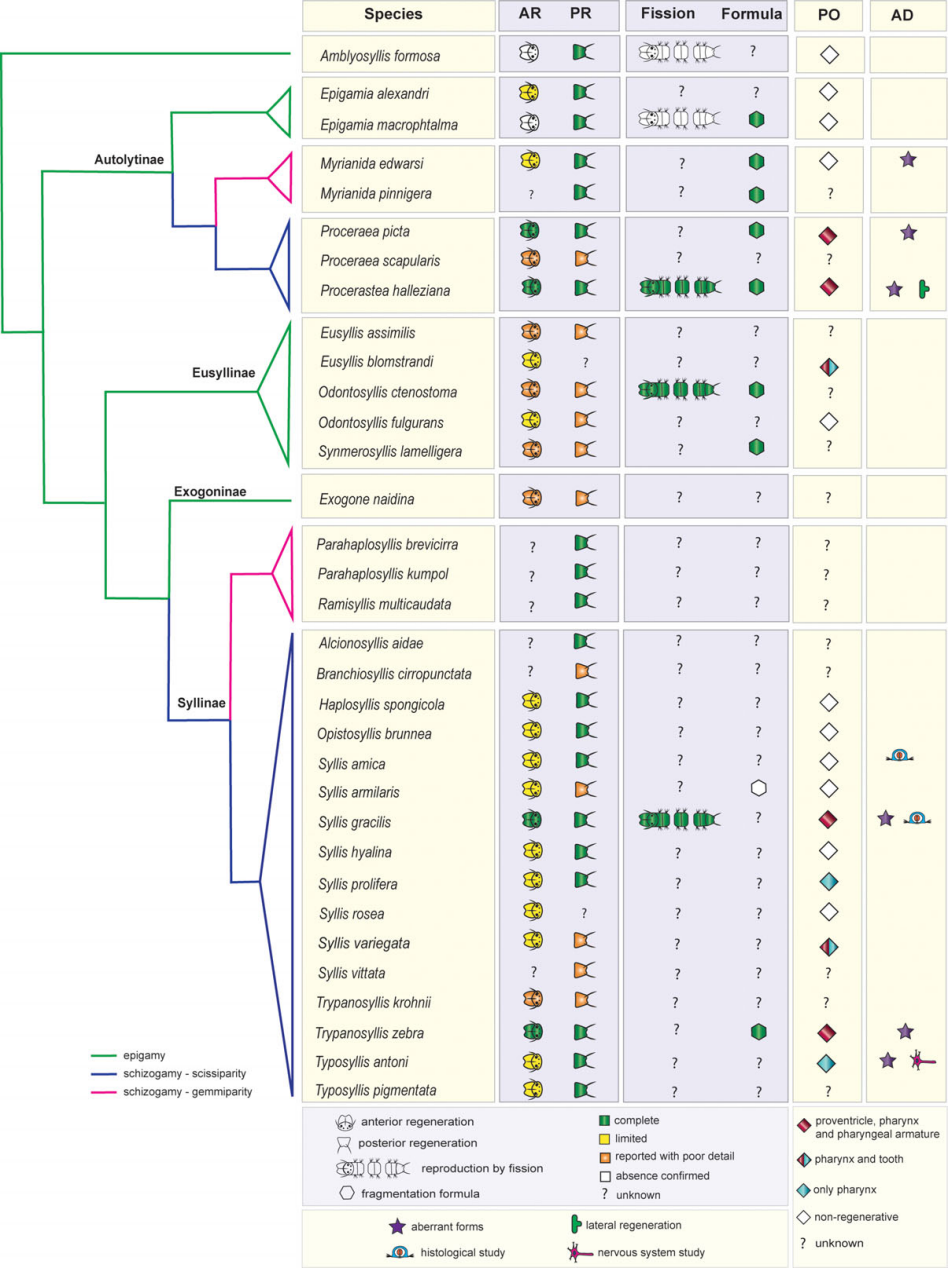
Distribution of some regenerative abilities and fragmentation in Syllidae. Regeneration data are based on reports of regenerating species and experimental studies (see Table 2). Presence and absence of regeneration was considered following, in general, categories by Zattara and Bely (2016), with some modifications: (1) axial regeneration absent (not even small portions of terminal tissue regenerate), (2) type I axial regeneration (caps can be regenerated after partial removal of these), (3) type II axial regeneration (caps, one or more segments and part of digestive tube can be regenerated), and (4) type III axial regeneration (like type II, but all digestive tube can be regenerated). Limited regeneration comprises inability to restore complete organs and extent comparable with the lost body before bisection, which includes type I and II axial categories. Type III was considered as presence of complete anterior or posterior regeneration. As suggested by Aguado, Glasby et al. (2015) and Aguado, Helm et al., (2015), *Syllis* and *Typosyllis* names remain as they were originally described. See Table 1 for data covering the information in this figure. AD, additional information; AR, anterior regeneration; PO, pharyngeal organ regeneration; PR, posterior regeneration. Phylogeny and distribution of reproductive modes following the most recent hypotheses (Aguado et al., 2012; Aguado, Glasby et al., 2015; Aguado, Helm et al., 2015)

**Figure 4:**
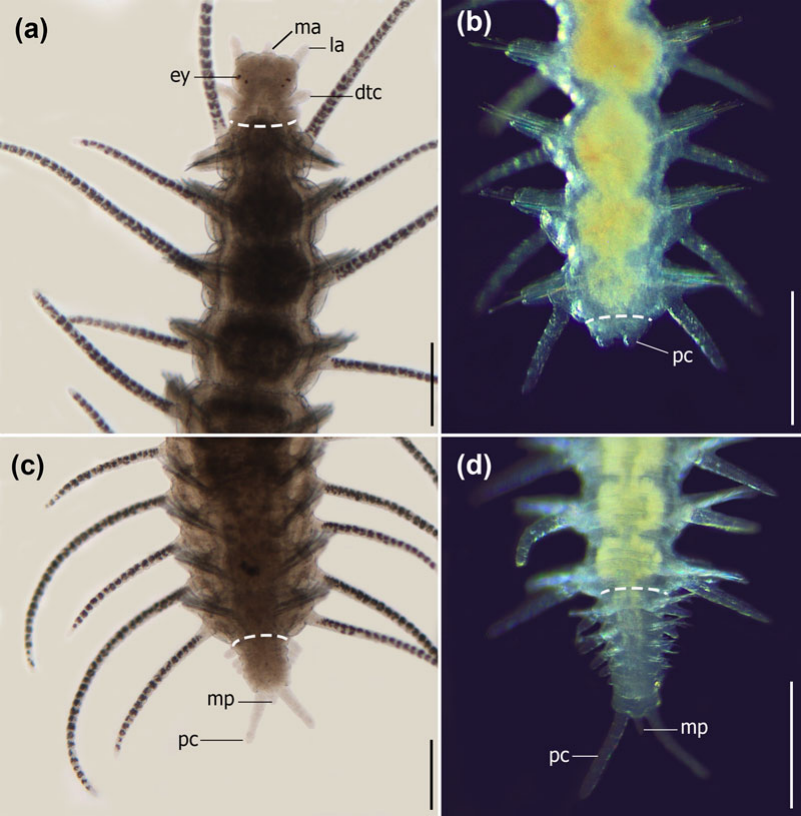
Light microscope pictures of two regenerating species of syllids, *Typosyllis antoni* (A and C) (pictures by Michael Weidhase reproduced by permission) (Aguado, Glasby et al., 2015; Aguado, Helm et al., 2015), and *Syllis* sp. (B and D). (A) Anterior regeneration, 6 days after dissection (dad). (B) Early stages of posterior regeneration, pygidium and cirri present. (C) Posterior regeneration in 6 dad. (D) Growth stage of posterior regeneration. Dashed line represents the dissection region. All pictures are dorsal view. dtc, dorsal tentacular cirrus; ey, eye; ma, median antenna; mp, median papila; pc, pygidial cirrus; la, lateral antenna. Scale bar 250 μm

Currently, syllids known to be able to regenerate belong to Autolytinae, Syllinae, Eusyllinae, Exogoninae, and Amblyosyllis (Figures 3,4A−D and Table 1). No information is available for Anoplosyllinae or *Perkinsyllis* species. Despite that, all schizogamous species are supposed to regenerate at least posteriorly. In general, syllids regenerate from fragments containing the pygidium or prostomium, or from isolated midbody segments. For instance, a fragment comprising the prostomium and two more chaetigers in *Trypanosyllis zebra* is able to regenerate up to 24 segments (Okada, 1929). From an isolated segment, *Amblyosyllis formosa* was observed regenerating posteriorly the pygidium and three segments (Abeloos, 1952). Autolytines are able to regenerate anteriorly and posteriorly including short fragments with only two, three, and four segments (Allen, 1923, 1927a, 1927b). Fragments with two segments (chaetiger 12−13) of *Procerastea halleziana* can regenerate anteriorly up to seven segments and prostomium, and posteriorly the pygidium plus four segments (Allen, 1923). In contrast, most of the sylline species, such as *Syllis amica* (Boilly, 1967d), *T. antoni* (Weidhase, Beckers et al., 2016) and *Odontosyllis fulgurans* (Okada, 1929), revealed a limited anterior regeneration demonstrated, for example, by the lack of a new proventricle, pharynx, or pharyngeal tooth (Figures 3, 4A, Table 1).

Regeneration in syllids occurs by an epimorphic process, but morphallaxis is also observed (see Section 6) (Boilly, 1969; Delye, 1962; Özpolat & Bely, 2016; Weidhase et al., 2017; Zattara & Bely, 2016). After amputation, at first an invagination is formed at the edge of the wound; after about 1–3 days the wound heals, and then dedifferentiation or blastema formation is initiated (Boilly, 1962b, 1967a, 1967c; Weidhase, Beckers et al., 2016; Weidhase et al. 2017). The next steps are blastema growth, re‐differentiation, tissue remodeling, and resegmentation (Boilly, 1968a, 1968b; Wissocq, 1970a, 1970b) (see Section 5). The total regeneration time is variable among species, and there is a lack of detailed information about how long the process takes (Table 1). In comparison with other species the development of the prostomium and brain of *T. antoni* is relatively fast (Weidhase et al., 2017) (Table 1). Similarly, *P. halleziana* and *S. amica* also show an early appearance of the prostomium 4 and 5 days after amputation, respectively (Allen, 1923; Boilly, 1967a).

## 3 REGENERATION AND SEXUAL REPRODUCTION IN SYLLIDAE

Syllids can reproduce sexually by epitoky, a process in which adult individuals undergo a metamorphosis before spawning (Aguado et al., 2012; Franke, 1999). Epitoky can be subdivided into two different modes: epigamy and schizogamy (Aguado et al., 2012; Franke, 1999; Nygren & Sundberg, 2003). In epigamy, the sexually mature organisms modify the body by developing a hypertrophied cephalic sensory apparatus and swimming notochaeta in the midbody‐posterior segments. After spawning the adult organisms usually die (Aguado et al., 2012; Schroeder & Hermans, 1975). In schizogamy, the modified segments additionally develop a head and become sexual units called stolons that can be detached from the adult body (Figure 2) (Aguado, Glasby et al., 2015; Aguado et al., 2012). Eventually, the stolons die after spawning and/or release of juveniles (for species in which brooding occurs), while the adult body regenerates new posterior segments and is able to reproduce again (Figure 2) (Aguado, Helm et al., 2015; Franke, 1999). Sometimes regeneration of a new posterior end precedes the release of stolons (Álvarez‐Campos, Martín, & Aguado, 2013; San Martín, Hutchings, & Aguado, 2008). Therefore, in the case of schizogamous syllids regeneration represents part of the normal reproductive process (Figure 2D).

**Figure 5:**
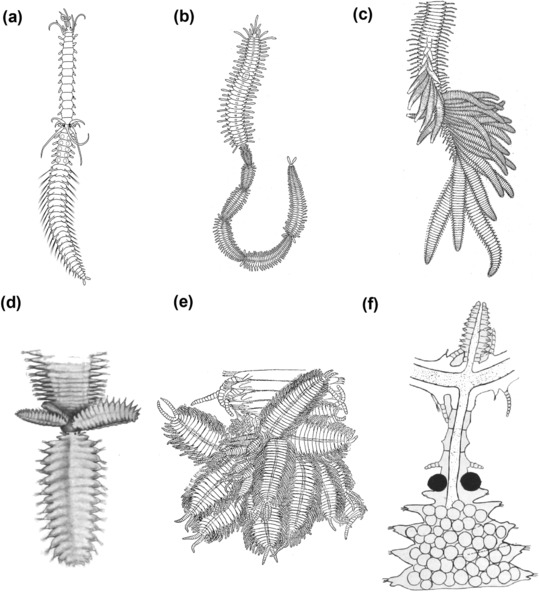
Different types of schizogamy: (A) scissiparity and (B)–(F) gemmiparity. (A) Scissiparous species *Proceraea cornuta* modified after Okada (1933a); a single stolon is produced per stolonization event. (B) Sequential gemmiparity in *Myrianida* sp., modified after Okada (1933b). (C). Successive budding in *Trypanobia asterobia*, modified after Okada (1933b). (D) Collateral budding in *Trypanosyllis crosslandi* Potts, 1911, modified after Potts (1911). (E) Collateral budding in *Trypanedenta gemmipara* (Johnson, 1901), modified after Johnson (1902). (F) Branching gemmiparity: branches and stolon in *Syllis ramosa*, modified after McIntosh (1885). All modified figures are in the public domain

Additionally, there are two types of schizogamic reproduction named gemmiparity and scissiparity (Figures 2 and 5). In gemmiparity several successive stolons are produced simultaneously, while in scissiparity only one stolon is produced at a time (Nygren & Sundberg, 2003). In scissiparity, the stolons result from the modification of existing segments, whereas in gemmiparity the stolons are developed de novo, i.e., new segments are generated to form the stolons (Franke, 1999; Schroeder & Hermans, 1975). Scissiparity is also found, although with some differences, in other groups of annelids such as Eunicidae (Rouse & Pleijel, 2006); in contrast, gemmiparity is unique to syllids (Aguado, Glasby et al., 2015).

The phylogenetic relationships within Syllidae are well resolved (Aguado et al., 2007, 2012; Aguado, Glasby et al., 2015; Aguado, Helm et al., 2015) and thus the evolution of certain features and processes, such as reproduction, can be traced. Epigamy (in Amblyosyllis, Eusyllinae, Exogoninae, Epigamia, and Perkinsyllis) is hypothesized to be the plesiomorphic condition in Syllidae, and schizogamy has evolved separately in Syllinae and Autolytinae (Figure 3) (Aguado et al., 2007, 2012; Nygren & Sundberg 2003). Regarding the schizogamous modes, gemmiparity is derived from scissiparity in two independent clades: *Myrianida* (Autolytinae) and in some genera of the so‐called “ribbon” clade (Aguado, Glasby et al., 2015) (within Syllinae), such as *Parahaplosyllis*, *Ramisyllis*, *Trypanedenta*, and *Trypanobia* (Aguado et al., 2012; Aguado, Glasby et al., 2015).

Schizogamous species seem to show higher anterior regenerative potential (Weidhase et al., 2017) (Figure 3, Table 1). On the other hand, the regeneration abilities in epigamous species are frequently limited, such as in Eusyllinae (Boilly, 1961; Okada, 1929), autolytines of genus *Epigamia* (Allen, 1927a), and *A. formosa* (Abeloos, 1952). Only few details regarding the regeneration in Exogoninae are available (Figure 3, Table 1).

Interestingly, regeneration studies of stolonizing individuals of *Proceraea picta* revealed the regression of stolon and different types of regenerated heads (Durchon & Wissocq, 1962, 1964; Okada, 1929). The experiments consisted of bisecting the animals in segments 13 or 14, where usually the stolon head is formed (Durchon & Wissocq, 1964). When this bisection is made in early stages of stolonization, in which a differentiation of a brain, eyes and appendages occurs, the posterior piece regenerates an adult head at the level of the stolon head (Okada, 1929) while the anterior piece regenerates a new tail, and the previous stolon head is completely regressed (Durchon & Wissocq, 1962, 1964). This regression depends on the section region. Dissections closer to the stolon head promote the regression, whereas dissections closer to the posterior end do not interfere in stolon formation (Durchon & Wissocq, 1962). These results point out that regeneration inhibits stolonization depending on the distance of the bisection from the stolon head (Durchon & Wissocq, 1962, 1964). Similar findings were also described in *P. leidyi*, a clitellate annelid that reproduces by paratomic fission, where amputation after the fission zone could provoke its resorption (Zattara & Bely, 2013).

Multiple SAZs are observed during stolonization of gemmiparous species, such as those of autolytine *Myrianida* and the syllines *Trypanobia, Parahaplosyllis, Trypanosyllis*, and *Trypanedenta* (Figure 5B–F) (Delye, 1962; Okada, 1933b; Potts, 1913). *Myrianida* stolonizes by sequential gemmiparity, in which stolons are generated in the same antero‐posterior axis of the body (Figure 5B) (Aguado, Glasby et al., 2015; Okada, 1933b). *Trypanobia asterobia*, for example, stolonizes by successive gemmiparity (Figure 5C) (one stolon per segment, projected from the ventral side) (Aguado, Glasby et al., 2015; Okada, 1933b). Species of *Parahaplosyllis*, *Trypanedenta* and *Trypanosyllis* can stolonize by collateral budding (Figure 5D–E) (stolons are projected in different directions from a specific segment). Additionally, a branching gemmiparity was reported for *Syllis ramosa* (McIntosh, 1879) and *Ramisyllis multicaudata* (Glasby, Schroeder, & Aguado, 2012) (Figure 5F). The body of these species is clearly ramified consisting of several branches and each terminal branch has a pygidium (Aguado, Glasby et al., 2015; Glasby et al., 2012; Schroeder, Aguado, Malpartida, & Glasby, 2017). These species develop stolons in the posterior end of different terminal branches (Aguado, Glasby et al., 2015; Glasby et al., 2012). *Parahaplosyllis*, *Trypanosyllis*, and *Trypanobia* are phylogenetically closely related with *R. multicaudata*, within the “ribbon clade” (Aguado, Glasby et al., 2015). Therefore, ribbon‐clade species share the ability to develop multiple SAZs and stolons with antero‐posterior axes different from the original body. This process apparently violates the bilateral symmetry in annelids, maintained by the activity of only one SAZ (Balavoine, 2014; Bely, 2006). Formation of multiple SAZs during stolonization appears to be restricted to gemmiparous species.

## 4 REGENERATION AND ASEXUAL REPRODUCTION IN SYLLIDAE

In annelids, asexual reproduction is always accomplished by subdivision of the body and regeneration of missing parts (Schroeder & Hermans, 1975). This process is widely known as fission and can occur in two different ways: fission or architomy, in which physical separation precedes the development of new tissues; and paratomy, in which new tissues are developed prior to physical separation (Zattara & Bely, 2016). Phylogenetic analyses in annelids suggest that fission has repeatedly evolved by co‐option of regenerative abilities and the presence of anterior regeneration is necessary to evolve fission (Zattara & Bely, 2016).

**Figure 6:**
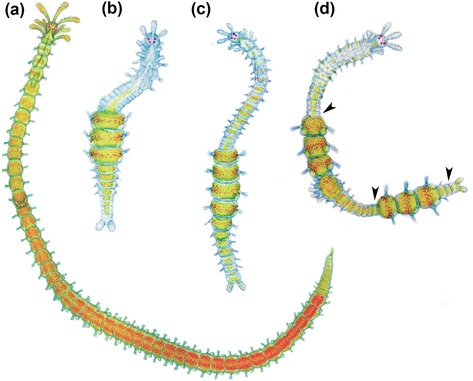
Regeneration of *Procerastea halleziana*. (A) Adult specimen starting stolonization. (B) Specimen regenerating from three original segments of midbody. (C) Specimen regenerating from four original segments of midbody after defective fragmentation. (D) Aberrant specimen regenerating after fragmentation, there is a regenerate between two fragments; arrows point at putative SAZs. Modified from Allen (1923). All modified figures are in the public domain

**Figure 7:**
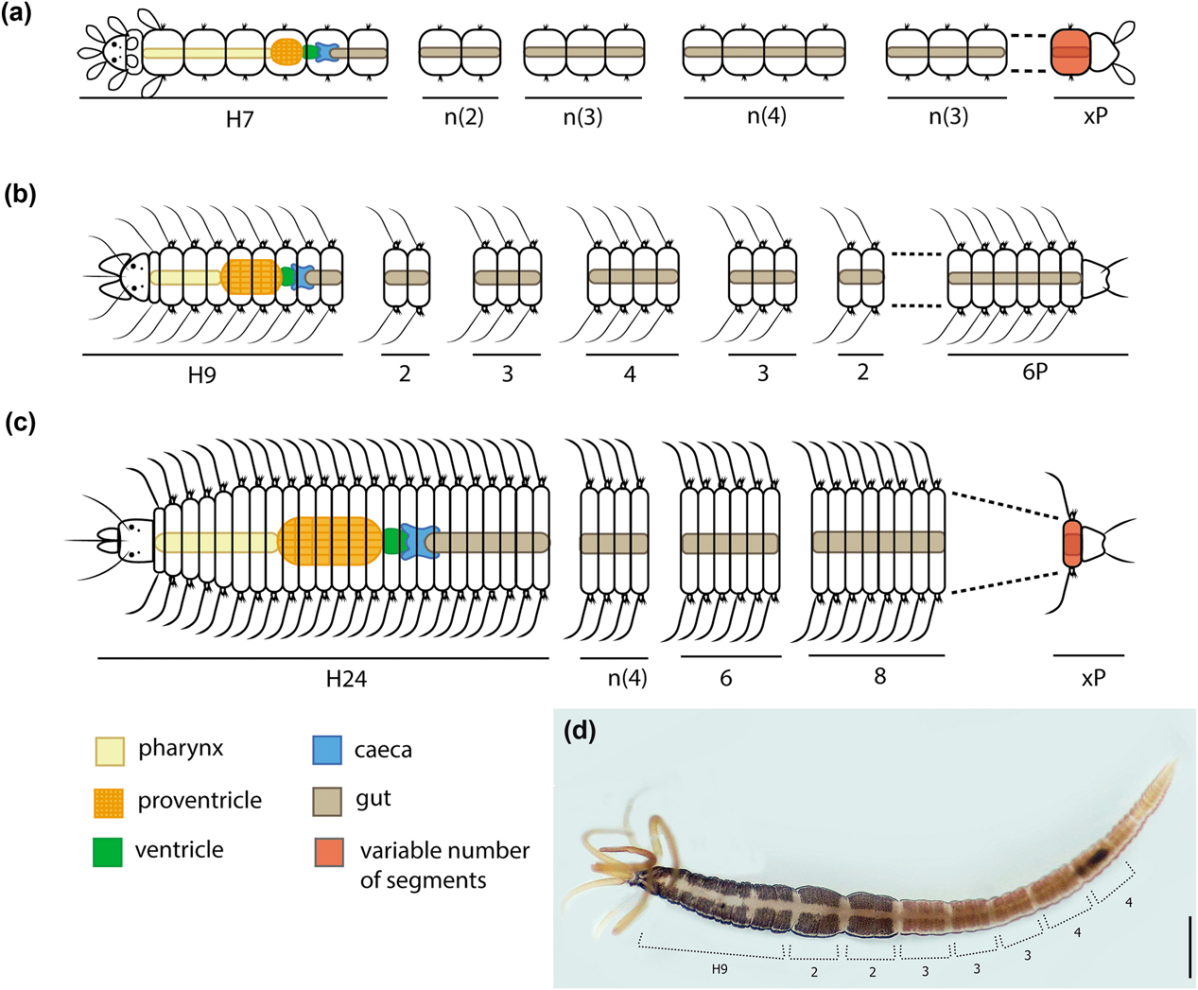
Fragmentation series in species of three subfamilies of Syllidae. (A) Autolytinae, *Procerastea halleziana*, after Allen (1923, 1927a, 1927b). (B) Eusyllinae, *Synmerosyllis lamelligera* (Allen, 1927b). (C) Syllinae, *Trypanosyllis zebra*, after Okada (1929). (D) Autolytinae, *Procerea picta*, collected from Ferrol, Galicia, Spain; the method of intercalated addition of distilled water and seawater was performed with this specimen, following Allen (1923). Regions of constrictions are marked indicating their number of segments. The series obtained with the constriction regions is comparable to the fragmentation series described for this species, following the color pattern (Allen, 1927a; Okada, 1929). Formula characters: H, head; *n*, variable number of fragments; *x*, variable number of segments; P, pygidium. Scale bar 500 μm

True asexual reproduction seems to be extremely rare in syllids (Franke, 1999). Some few species with high regenerative abilities may reproduce asexually by fission, such as *P. halleziana* (Figure 6) (Allen, 1923, 1927a, see below) and *S. gracilis* (Mesnil & Caullery, 1919) (Figure 3). This reproductive mode implies the development of new individuals from fragments of the stock organism that are capable of regenerating new anterior and/or posterior ends (Allen, 1923, 1927a, 1927b; Boilly & Thibaut, 1974; Okada, 1929) (Figures 6, 7).

During the first decades of the 20th century, several interesting investigations were carried out regarding a possible established program of fission and regeneration in certain species of syllids. Allen (1923) found that *P. halleziana* revealed some exact fission areas in the body, after experimental induction of fission by wetting the animals alternately with distilled water and seawater (Figures 6, 7, Table 2). As a result, the pattern of fission areas in *P. halleziana* could be described mathematically by a “fragmentation formula”: H7 + 2 + 2 + 2 + 3 + 3 + 3 + 4 + 4 + 4 + 4(+4) + 3 + 3 + 3 + 3 + *n*(+3) + *x*P, where the numbers indicate the segments of each fragmented piece (H, head; P, pygidium) (Allen, 1923) (Figure 7A, Table 2). The possible existence of specific fragmentation formulae stimulated a series of experiments in different groups of syllids, especially in Autolytinae (Allen, 1927a, 1927b; Okada, 1929). Okada (1929) found that the addition of a KCl solution induced this fragmentation as well. The contact with the solution provoked an unusually strong contraction of the longitudinal muscles of specific segments (Okada, 1929). Both methods (distilled/salted water; KCL solution) revealed that the animals fragmented in specific areas of the body (Figure 7, Table 2). However, the fragmentation formula of each species may vary depending on the method used, either with distilled water or KCl solution (Okada, 1929).

**Table 2:** Fragmentation series of syllids

Species	Formula	Reference
*Procerastea halleziana*	H7+2+2+2+3+3+3+4+4+4+4(+4)+3+3+3+3+*x*(+3)+*n*P	Allen, 1923
*Myrianida prolifera*	H7+2+2+2+3+3+3+4+4+4+4+4+3+3+4+4+4+25P	
	H13+3+3+3+4+4+*x*(+3)+12P	Allen, 1927a
*Myrianida inermis*	H9+2+2+3+3+3+4+*n*S	Allen, 1927a
	H9+2+2+3+3+3+4+4+4+14P	
	H11+2+3+3+3+4+4+4+4+4+3+3+7P	
*Myrianida edwarsi*	H7+2+2+2+3+3+3+4+4+*n*S	Allen, 1927a
	H11+2+3 + 3+3+4+4+4+3+*n*S	
*Myrianida pinnigera*	H13 +3 +3+3+4 +4 +4 +3 +3 +4 +4 +4 +4 +4 +4 +3 +3+3+(…)+*n*P	Okada, 1929
*Proceraea picta*	H9+2+2+3+3+3+4+4+4+4+4+3+3+4+4 +4+4+4+4+1+3+5P	Allen, 1927a
*Epigamia macrophtalma*	H7+2+2+2+3+3+3+4+4+4+4+4+3+3+4+4+4+4+4+4+4+4+4+11P	Allen, 1927a
	H7+2+2+2+3+3+3+4+4+4+4+4+3+3+4+4+4+4+4+4+4+4+4+6P	
*Synmerosyllis lamelligera*	H9+2+2+3+3+3+4+3+4+2+2+6P	Allen, 1927b
*Nudisyllis divaricata*	H9+4+3+3+3+4+5+4+3+4+4+4+5P	Okada, 1929
*Trypanosyllis zebra*	H24+4+4+4+6+8+4+6+4+18+6+6+12+3+4+39P	Okada, 1929
	H24+4+4+4+6+8+4+6+4+18+6+6+12+3+4+*n*S	

The fragment with the prostomium is represented by H and the number of segments, and the last fragment, with the pygidium is represented by the number of segments and P, adapted after Allen (1923). H, head; P, pygidium; *n*S, number of segments corresponding to stolon; *n*P, variable number of segments plus pygidium.

According to these studies, the first fragment (with prostomium) usually breaks in an odd number of segments and it is usually longer than the rest (Figure 7A–C, Table 2). In autolytines and eusyllines, the number of segments in the anterior fragments is usually 7, 9, 11 or 13 (Allen, 1927a, 1927b; Okada, 1929). Generally, posterior fragments vary in number of segments and in a stolonizing individual the break occurs at the level where the stolon head is developed (Allen, 1927a; Okada, 1929). Furthermore, some species exhibited high variation in the formula, depending on the presence or absence of stolon (Allen, 1927a). Interestingly, the fragmentation formulae of *Proceraea picta* and *Epigamia macrophtalma* coincide with the color pattern found in the body surface of these species (Allen, 1927a). We have repeated the experiment of fragmentation induction with distilled water in a specimen of *P. picta*. Although the animal did not break, constrictions appeared in the same fragmenting areas previously described coinciding with its color pattern (Figure 7D).

Ten species are known to show a fragmentation formula, including those that do not reproduce by fission, i.e., species in which the fragments are unable to regenerate completely (Table 2). Fission can be an asexual reproductive mode easily confounded with non‐reproductive autotomy and post‐injury regeneration, especially from field observations (Zattara & Bely, 2016). The fragmentation formulae observed experimentally in syllids (Allen, 1923, 1927a; Okada, 1929) might indeed be the result of a stress induction and not a truly asexual reproductive process (Franke, 1999; Langhammer, 1928). The addition of distilled water could be the factor that provokes autotomy as a defensive strategy based on discard of part of the body.

## 5 ANTERIOR VERSUS POSTERIOR REGENERATION

Most of the studied species regenerate anterior as well as posterior ends of the body, with the exception of *A. formosa* and *E. macrophtalma* that are not able to regenerate anteriorly (Figure 3, Table 1). However, fragments of both these species can survive for some weeks without a regenerated head (Abeloos, 1952; Allen, 1927a). Interestingly, in *A. formosa*, a species with a fixed number of segments, the regeneration of the posterior end never exceeds the original number of segments before amputation (Abeloos, 1952). All remaining investigated syllids are able to redevelop a new anterior end, although this is limited to a regenerated prostomium and few segments with missing organs in many species, i.e., *Eusyllis blomstrandi* (Boilly, 1961), *O. fulgurans* (Saint‐Joseph, 1886), *Syllis prolifera* (Abeloos, 1950), and *T. antoni* (Aguado, Helm et al., 2015; Weidhase, Beckers et al., 2016; Weidhase et al., 2017) (Figure 3).

**Figure 8:**
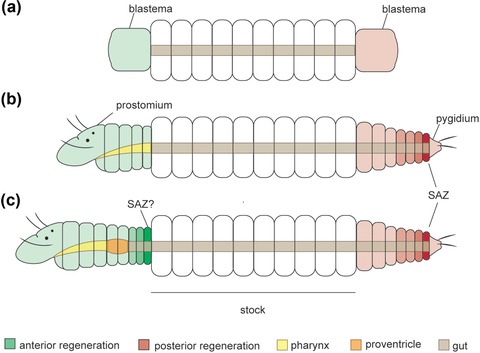
Schematic drawings of anterior and posterior resegmentation in syllids. (A) Blastema formation; generalized scheme for Syllidae. (B) Anterior and posterior pattern of resegmentation based on eusyllines and syllines (Aguado, Helm et al., 2015; Boilly, 1961, 1967c; Weidhase, Beckers et al., 2016; Weidhase et al., 2017). (C) Anterior and posterior patterns of resegmentation based on the autolytine *Procerastea halleziana* (Allen, 1923). Establishment of posterior SAZ (B), (C). Anterior regeneration: a SAZ is not detectable (B) or takes place in the region immediately attached to the stock segment (C). SAZ, segment addition zone

Anterior resegmentation and posterior resegmentation involve different processes that vary among species (Figure 8). Most authors observed that posterior regeneration involves the establishment of a SAZ, immediately before the pygidium (Allen, 1923; Weidhase, Beckers et al., 2016) (Figure 8B, C). Concerning anterior resegmentation, two patterns were observed in different species of syllids. In most syllids the blastema grows and differentiates into a prostomium (at first) and regenerated segments, and a SAZ is transient or not detectable (Figure 8B) (Aguado, Helm et al., 2015; Boilly, 1961, 1965a; Weidhase, Beckers et al., 2016, Weidhase et al., 2017). However, in some cases the anterior regeneration seems to bear a SAZ, because segments are regenerated by sequential addition from the original segment of the stock (Figure 8C) (Aguado, Helm et al., 2015; Allen, 1923). Whereas the first pattern is widely recognized in annelid regeneration (Balavoine, 2014), the second is unusual, reported only for the syllids *P. halleziana* and (in some instances) *T. antoni* (Aguado, Helm et al., 2015; Allen, 1923). In this case, fragments of the midbody of *P. halleziana* can establish two SAZs (anterior and posterior), in which segment addition occurs following the posterior−anterior direction (Allen, 1923) (Figure 8C). As is well known for nereidids, posterior growth during juvenile development involves similar genetic pathways (as evidenced by similar gene expression patterns) to those occurring in the SAZ during posterior regeneration (Balavoine, 2014; Prud'homme et al., 2003). Unfortunately, these animals do not regenerate the anterior body. Whether anterior growth could also share similar mechanisms to juvenile growth is a topic that still needs to be clarified.

Boilly (1961) and Okada (1929) showed that the extent of regeneration depends on the region of dissection. Specimens of *Eusyllis blomstrandi* failed to regenerate anteriorly or died when dissected in the most posterior part of the body (Boilly, 1961). A similar limitation was observed in the posterior regeneration of *Myrianida edwarsi*, in which the anterior end needed a certain number of segments to regenerate a new tail (Okada, 1929). In general, posterior regeneration usually produces more new segments than anterior regeneration, with the exception of *S. gracilis* that is able to regenerate anteriorly up to 18 segments and posteriorly up to eight segments (Boilly & Thibaut, 1974; Mesnil, 1901) (Table 1).

Regarding the speed of regeneration, a new pygidium appears faster than a new prostomium according to the literature, even though huge differences in the pace of regeneration have been recorded (Table 1). The prostomium appears 4–35 days after dissection (dad) in the investigated syllid species (Allen, 1923; Boilly, 1967a, 1967c; Franke, 1980, 1983; Weidhase, Beckers et al., 2016; Weidhase et al., 2017) (Table 1). *S. prolifera* takes the longest time to regenerate a new prostomium (35 dad) (Franke, 1983), whereas *T. antoni* and *P. halleziana* need no more than 4 dad (Aguado, Helm et al., 2015; Allen, 1923; Weidhase, Beckers et al., 2016; Weidhase et al., 2017). The maximum number of regenerated segments recorded is 29 in *P. halleziana*. The appearance of pygidium varies between 3 and 9 dad (Allen, 1923; Boilly, 1967a, 1967c; Delye, 1962; Franke, 1980, 1983; Weidhase, Beckers et al., 2016; Weidhase et al., 2017) (Table 1). *P. halleziana* and *T. zebra* regenerate, respectively, 35 and 24 segments posteriorly; those are the highest number of regenerated segments recognized in syllids (Allen, 1923; Delye, 1962) (Table 1) .

## 6 CELLS, TISSUES AND ORGANS INVOLVED

Early stages of regeneration including cell dynamics, histogenesis, and renewal of organs were the focus of some investigations in syllids. The knowledge about cell dynamics and histogenesis was mainly provided by studies on *S. amica* (Boilly, 1962a, 1965, 1967a, 1967b, 1967c, 1967d, 1968a, 1968b; Wissocq, 1970a, 1970b). Punctual observations about organ regeneration are available (Allen, 1923; Boilly, 1967a; Boilly & Thibaut, 1974; Delye, 1962; Okada, 1929; Weidhase, Beckers et al., 2016; Weidhase et al., 2017).

Anterior and posterior regeneration appear to share similar mechanisms of cell proliferation and cells forming the regenerated tissues seem to have the same origin (Boilly, 1962a, 1962b, 1965a, 1965b, 1967a, 1967b, 1967c, 1967d, 1968a, 1968b; Boilly & Thibaut, 1974). The first signs of re‐establishment of tissues in the regeneration of *S. amica* are an invagination and retraction of the intestine (Boilly, 1967a). Both anterior and posterior fragments exhibit wound healing, followed by blastema formation by bulging of the scarring epidermis in 2–3 dad (Boilly, 1962b, 1967a). As a result of several studies, three phases of cell dynamics during regeneration were observed: (1) cellular dedifferentiation, loss of the elements that configure the characteristics of each cellular type; (2) activation and transformation of the regenerative cells related to intense synthesis of RNA; and (3) differentiation, characterized by an acquisition of a new structure and specific proteins (Boilly, 1962a, 1962b, 1965a, 1965b, 1967a, 1967b, 1967c, 1967d, 1968a, 1968b).

Blastema formation is the main characteristic of the dedifferentiation stage. The cells involved in blastema formation and renewing tissues derive from the last segment preceding the section site (Boilly, 1962a, 1962b, 1965). In contrast to clitellates, migratory regenerative cells have so far not been found in Syllidae (Boilly, 1967a). A quantitative antero‐posterior gradient has been observed in blastemal tissues of anterior regeneration in *M. edwarsi* (Okada, 1929). The more posterior the dissection region, the smaller blastema, and the total quantity of tissue derived from blastema is reduced (Berrill, 1952; Okada, 1929). The activation stage is supposed to be dependent on nervous fibers close to the wound region, and it begins once the signals of activation appear in the cells near the nerve chain (Boilly, 1967a, 1967b). This process does not occur immediately after section, due to the time required to heal the axon that elongates towards the microenvironment of the blastema and stimulates regeneration (Boilly, 1967a; Boilly, Faulkner et al., 2017). Neuropeptides, neurotransmitters, growth factors, and morphogens were suggested as mediators in this activation process (Boilly, Boilly‐Marer et al., 2017). Finally, during the differentiation stage, the regenerative cells experience changes in cytoplasmic elements to acquire specialized functions, e.g., muscular and epidermal cells (Boilly, 1967a, 1968b). During this process, ribosomes are abundant and proteins associated with cell proliferation and differentiation are clearly observed (Boilly, 1968b).

The available information about the regeneration of different organs and tissues is limited in syllids. Most detailed descriptions about digestive tube, muscular, and nervous tissue regeneration are available from former studies in *S. amica* and, more recently, *T. antoni* (Boilly, 1962a, 1962b, 1965a, 1965b, 1967a, 1967b, 1967c, 1967d, 1968a, 1968b; Weidhase, Beckers et al., 2016; Weidhase et al., 2017; Wissocq, 1970a, 1970b).

In *S. amica*, the digestive tube regenerates supposedly from ectodermal, mesodermal, and endodermal tissues (Boilly, 1967c, 1969). The pharynx has ectomesodermal origin and the intestinal epithelium originates from ectodermal and mesodermal regenerative cells (Boilly, 1967c, 1969). However, these studies employed limited methodologies to trace cell lineage during regeneration in *S. amica*; hence these results should be verified with state‐of‐the‐art methods. Curiously, specimens that after anterior amputation lack part of the pharynx or proventricle regenerate an intestine posteriorly, instead of the missing part of the pharynx or the proventricle (Boilly, 1967c). The regeneration of pharynx, proventricle, or, more occasionally, some anterior appendages is missing or incomplete in many syllids (Abeloos, 1950; Boilly, 1961; Michel, 1909b; Okada, 1929; Weidhase, Beckers et al., 2016) (Table 1). A few species restore the proventricle and the pharyngeal armature (tooth or trepan), such as *P. halleziana* (Allen, 1923), *T. zebra* (Delye, 1962), and *S. gracilis* (Boilly & Thibaut, 1974; and personal observation) (Figure 3).

Regeneration of the nervous system occurs by growth and elongation of the remaining nerve fibers (Weidhase et al., 2017). The nervous system of *T. antoni* begins to regenerate anteriorly during the blastema stage (2 dad) with an ingrowth of neurites originating in the remaining ventral nerve cord, and then neurites form loops in 4 dad together with the nerve cord elongation and development of a new brain (Weidhase et al., 2017). In *S. amica*, the ventral nerve cord was also observed in an elongation process from 3 dad and the prostomium was completely differentiated in 7 dad (Boilly, 1967a). The nervous system at the posterior end of *T. antoni* regenerates in a similar way, but the blastema is formed in 3 days, and a day after a new pygidium with cirri is present (Weidhase et al., 2017). This process is comparable to the posterior regeneration of *S. amica*, differing only in the appearance of the pygidium at 9 dad (Boilly, 1967a).

## 7 THE ROLE OF THE PROVENTRICLE DURING REGENERATION

Several studies in schizogamous species focused on the triggers of the stolonization process. Usually, experiments with removal of the proventricle indicated a potential role of this structure during reproduction (Durchon, 1957, 1959; Franke, 1980, 1983; Franke & Pfannenstiel, 1984; Weidhase, Beckers et al., 2016). Posterior ends of fragments without proventricle seem to increase the production of male stolons, suggesting that the proventricle inhibits the stolonization and is involved in sexual determination (Durchon, 1957, 1959; Durchon & Wissocq, 1964; Franke & Pfannenstiel, 1984; Heacox & Schroeder, 1982; Weidhase, Beckers et al., 2016; Wissocq, 1966). Experiments in which the proventricle is removed in *S. prolifera* have shown that stolonization stops when the structure is re‐implanted, indicating that the proventricle may inhibit stolonization while promoting regeneration (Franke & Pfannenstiel, 1984). However, no glandular structure was found in the proventricle of *T. antoni*, and a suggestion is that structures associated with this organ (e.g., nervous system) might be responsible for the regulation of reproductive and regenerative processes (Weidhase, Beckers et al., 2016).

In effect, stolonization is favored over regeneration in an individual unable to regenerate the proventricle and vice versa. Similar results were described regarding the interactions between paratomic fission and regeneration in other annelids and flatworms (e.g., Child, 1903; Zattara & Bely, 2013). This tendency of mutual inhibition between regeneration and stolonization or fission operates probably by a comparable machinery.

## 8 ABERRANT FORMS AND BRANCHING PATTERNS

**Figure 9:**
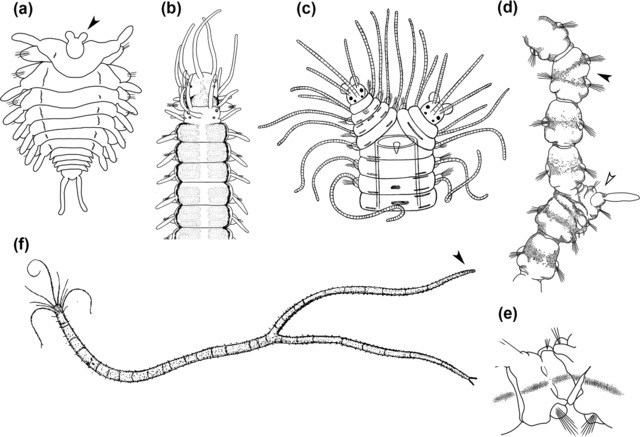
Aberrant forms in some species of Syllidae. (A) *Myrianida edwarsi*: arrow point at tail regenerated in anterior end after transversal dissection; modified after Okada (1929). (B) *Proceraea picta*: double head regenerated after transversal dissection between 12th and 13th segment, modified after Okada (1929). (C) *Syllis variegata*: specimen with bifurcated anterior end; modified after Andrews (1892). (D) *Procerastea halleziana*: filled arrow point at additional parapodium laterally projected in the right side of 16th segment, empty arrow point at lateral regeneration in 19th segment, dorsal view, and (E) ventral view of the regenerate; modified after Allen (1923). (F) *Myrianida ornata* specimen with bifurcated posterior ends: arrow point at abnormal tail without cirri; modified after Andrews (1892). All modified figures are in the public domain

Major phenotypic changes in syllids have been observed in experimental regeneration studies that report aberrations such as double heads and bifurcated forms (Aguado, Glasby et al., 2015; Allen, 1923; Okada, 1929; Weidhase, Beckers et al., 2016) (Figure 9A−F). An extraordinary case of heteromorphic regeneration happened in a specimen of *M. edwarsi* that restored a tail in the place of a head during anterior regeneration (Okada, 1929) (Figure 9A). Another related aberration is the development of double heads, as reported for *P. picta* after dissection between the segments 12 and 13 (Okada, 1929) (Figure 9B). Additionally, double heads were observed in stolons of *P. halleziana*, *S. amica*, *S. prolifera*, and *T. antoni*, during regeneration experiments of amputation with proventricle removal (Langhammer, 1928; Okada, 1934; Weidhase, Beckers et al., 2016).

Reports of bifurcated patterns in regeneration are known from *Syllis variegata* (Langerhans, 1879), *P. halleziana* (Allen, 1923; Langhammer, 1928), and *Myrianida ornata* (Andrews, 1891, 1892). A case of a bifurcated anterior end was observed in *S. variegata* under non‐experimental conditions by Langerhans (1879) (Figure 9C). The specimen probably had regenerated two heads after losing the original one (Andrews, 1892; Langerhans, 1879) (Figure 9C). Bifurcated tails are reported in a specimen of *M. ornata* found in nature (Figure 9F); the additional tail was interpreted as a lateral outgrowth (Andrews, 1891, 1892). A similar lateral projection was found in *P. halleziana* probably as a result of an error in the healing process after injury (Allen, 1923; Langhammer, 1928) (Figure 9D, E). Sometimes, bifurcated appendages such as antennae and pygidial cirri are observed during regeneration of several species (Durchon & Wissocq, 1964; Malaquin, 1893; Michel, 1909b).

Interestingly, as an error of the fragmentation process, *P. halleziana* exhibited a specimen with three regenerating regions, suggesting the activity of three different SAZs (Allen, 1923) (Figure 5D). Bifurcated or branching forms occurring in non‐gemmiparous species suggest that errors can arise during genetic control of regeneration and reproduction of syllids. It might be speculated that these kinds of “errors” may have led to the evolution of gemmiparity and branching species in Syllidae (Aguado, Glasby et al., 2015).

## 9 CONCLUSIONS AND FUTURE DIRECTIONS

Regenerative ability in syllids is linked to both sexual and asexual reproductive modes. During sexual reproduction by schizogamy, syllids regenerate the posterior body after release of a stolon, thereby being able to undergo another stolonization cycle. Asexual reproduction by architomic fission is known for species that can restore a complete body from fragments of the original individual. Stolonization in syllids and paratomic fission in annelids seem to share a regenerative mechanism in which mutual inhibition occurs, implying that the reproductive modes are comparable.

Studies in regeneration and fission facilitated another finding in syllid biology. Some species were able to split in specific areas during experiments of stress induction, including species that do not reproduce asexually. These events of fragmentation can be interpreted as an autotomic response rather than an architomic fission.

Considering whole‐body regeneration, syllids are able to regenerate anterior and posterior ends but often are limited in restoration of certain anterior organs (e.g., proventricle). Although this limited anterior regeneration is not complete (see Zattara & Bely, 2016), from a morphological point of view, the regenerating individuals are able to reproduce since the stolonization process is induced by proventricle removal. This organ seems to play an important role in reproduction and regeneration in syllids.

Some patterns regarding the speed and ability to regenerate segments and organs can be recognized in syllids. For instance, posterior regeneration is usually faster than anterior regeneration. Restoration of a new anterior end becomes easier when the bisection plane is close to the prostomium. Additionally, some species can renew the pharyngeal armature, and also show better overall anterior regenerative ability. Implications for ensuing studies can be a comparison of the genetic mechanisms during regeneration among syllids that reproduce by different modes.

The origin of regenerative cells in syllids has never been elucidated. Knowledge is limited to histological visualizations in different stages of regeneration, restricted to a single species. Studies of cell lineages during regeneration using fine‐scale methods and techniques of labeling and histology are missing.

Heteromorphic regeneration points out errors in the regenerative process that may have occurred and been established during the evolution of branching body patterns in syllids, such as those of *R. multicaudata* and *S. ramosa*. The occurrence of aberrant forms in stages of regeneration exemplifies an intriguing possibility of investigating the underlying genetic control processes. Therefore, syllid regeneration should be considered as a good model to study the evolution and development of body axes. At present, studies about gene expression patterns during regeneration or development in syllids are still missing. Emerging genomic methods will be useful to understand this process. By sequencing genomes and transcriptomes, genes involved in regeneration can be identified (Bhambri et al., 2017; Myohara et al., 2006; Nyberg et al., 2012), thereby opening up the possibility of a functional characterization, e.g., by CRISPR or RNAi methods (Boettcher & McManus, 2015). Different studies of annelid regeneration identified the expression of genes such as engrailed, wingless (Wnt), orthodenticle homologs, markers of germline cells (e.g. piwi, vasa and nanos), and Hox genes during regenerative processes (Bakalenko et al., 2013; Bely & Wray, 2001; Novikova et al., 2013; Özpolat & Bely, 2015; Prud'homme et al., 2003). Furthermore, morphogens (e.g., bone morphogenetic protein) and fibroblast growth factors might be involved in nervous activation of blastema formation (Boilly, Faulkner et al., 2017; Satoh, Makanae, Nishimoto, & Mitogawa, 2016; Takeo et al., 2013). These approaches are opportune to investigate the triggers of the regenerative response, the pathways that control growth and patterning of the new structures, and the re‐establishment of antero‐posterior polarity in syllids and other bilateral animals.

## CONFLICT OF INTEREST

The authors have no conflict of interest to declare.
